# Does the tendon retract in flexor hallucis longus muscle laceration? A cadaveric study

**DOI:** 10.1007/s00402-026-06378-1

**Published:** 2026-06-12

**Authors:** Murat Aydın, Yusuf Şahin, Hilal Yağar, Fatih Çiçek, Faruk Gazi Ceranoğlu, Ahmet Mert, Selim Çınaroğlu

**Affiliations:** 1 Büyük Anadolu Hospital Darıca, Kocaeli, Turkey; 2 Department Of Machine Theory and Dynamics, Faculty of Engineering, Niğde Ömer Halisdemir University, Niğde, Turkey; 3 Department of Orthopedics and Traumatology, Niğde Ömer Halisdemir University, Niğde, Turkey; 4 Department of Anatomy, Niğde Ömer Halisdemir University, Niğde, Turkey; 5 Private Pendik Yüzyıl Hospital, İstanbul, Turkey

**Keywords:** Biomechanics, Flexor hallucis longus, Tendon injury, Tensile force

## Abstract

**Introduction:**

Flexor hallucis longus (FHL) tendon injuries are common, particularly in open injuries caused by sharp objects. However, data regarding FHL tendon retraction following hallux-level FHL tendon lacerations remain limited. This study investigated the retraction and shortening of the FHL tendon using cadaveric models.

**Materials and methods:**

Six fresh-frozen below-knee amputated cadaveric feet (mean age: 65 years, range: 62–88) were utilized. FHL tendons were dissected, and lacerations were simulated at the hallux level. Tendons were subjected to various tensile forces (20–120 N) using a mechanical device. Force was measured in Newtons via a loadcell, and retraction distance was recorded in millimeters using an encoder. Data were summarized as mean ± standard deviation (SD), median, and quartiles.

**Results:**

The FHL tendon retracted gradually as applied force increased. An average retraction of 7 mm was measured at 20 N, reaching 21 mm at 60 N. Beyond 60 N (> 21 mm retraction), the other four toes also flexed. This occurs due to the anatomical connection between the FHL and flexor digitorum longus (FDL) tendons.

**Conclusions:**

Only slight retraction was observed in hallux-level FHL tendon lacerations. At most, the findings suggest that distal FHL lacerations may exhibit limited proximal retraction under the tested conditions, which may have implications for surgical planning. Understanding FHL biomechanics is essential for developing effective repair strategies. Further investigation into the clinical implications of these findings is warranted.

## Introduction

The flexor hallucis longus (FHL) muscle originates from the posterior fibula and interosseous membrane, and its tendon courses posterior to the ankle before passing along the plantar aspect of the foot [1–3]. The FHL tendon plays an essential role in flexion of the distal phalanx of the hallux and contributes to the final propulsive phase of gait, running, and jumping [3–5]. The tendons of the FHL and flexor digitorum longus (FDL) intersect in the medial plantar aspect of the foot and continue toward the level of the first tarsometatarsal joint. Thus, the hallux and lesser toes may also flex slightly during FHL muscle contraction [4, 5].

Tendon injuries can occur via any of the following three mechanisms: direct, indirect, or repetitive injuries [6–8]. Direct tendon injuries are caused by contusion, nonpenetrating blunt injury, or a cut with a sharp object such as broken glass due to accidents and sports injuries. In contrast, indirect or repetitive tendon injuries are caused by acute tensile overload and repetitive microtrauma, usually observed in overuse injuries [9].

Flexor tendon tears in the hand can lead to tendon retraction. One study reported that tendon repair is feasible within three weeks following flexor tendon injuries in the hand [10]. However, delayed repair can result in tendon end degeneration, fibrosis of the tendon sheath, formation of adhesions, and shortening of the muscle-tendon unit, thereby complicating both surgical repair and rehabilitation [11]. Despite this information regarding the hand flexor tendons, there is limited evidence regarding tendon retraction and optimal timing of surgery in cases of flexor tendon injuries in the foot. Therefore, this cadaveric biomechanical study aimed to quantify FHL tendon retraction after simulated hallux-level laceration under controlled tensile loading.

## Methods

### Study protocol

This study is an in vitro biomechanical human cadaver study It was conducted to determine the retraction distance changes depending on the forces applied to the muscle when the tendon of the FHL is lacerated or its integrity is compromised. It was approved by the non-interventional ethics committee of the Niğde Ömer Halisdemir University with approval number 2023/13.

This study was conducted on six fresh frozen below-knee amputated male foot cadavers with an average age of 65 (62–88), which were obtained with the support of Scientific Research Projects of Niğde Ömer Halisdemir University. 

### Preparation of materials for measurement

**Fig. 1:**
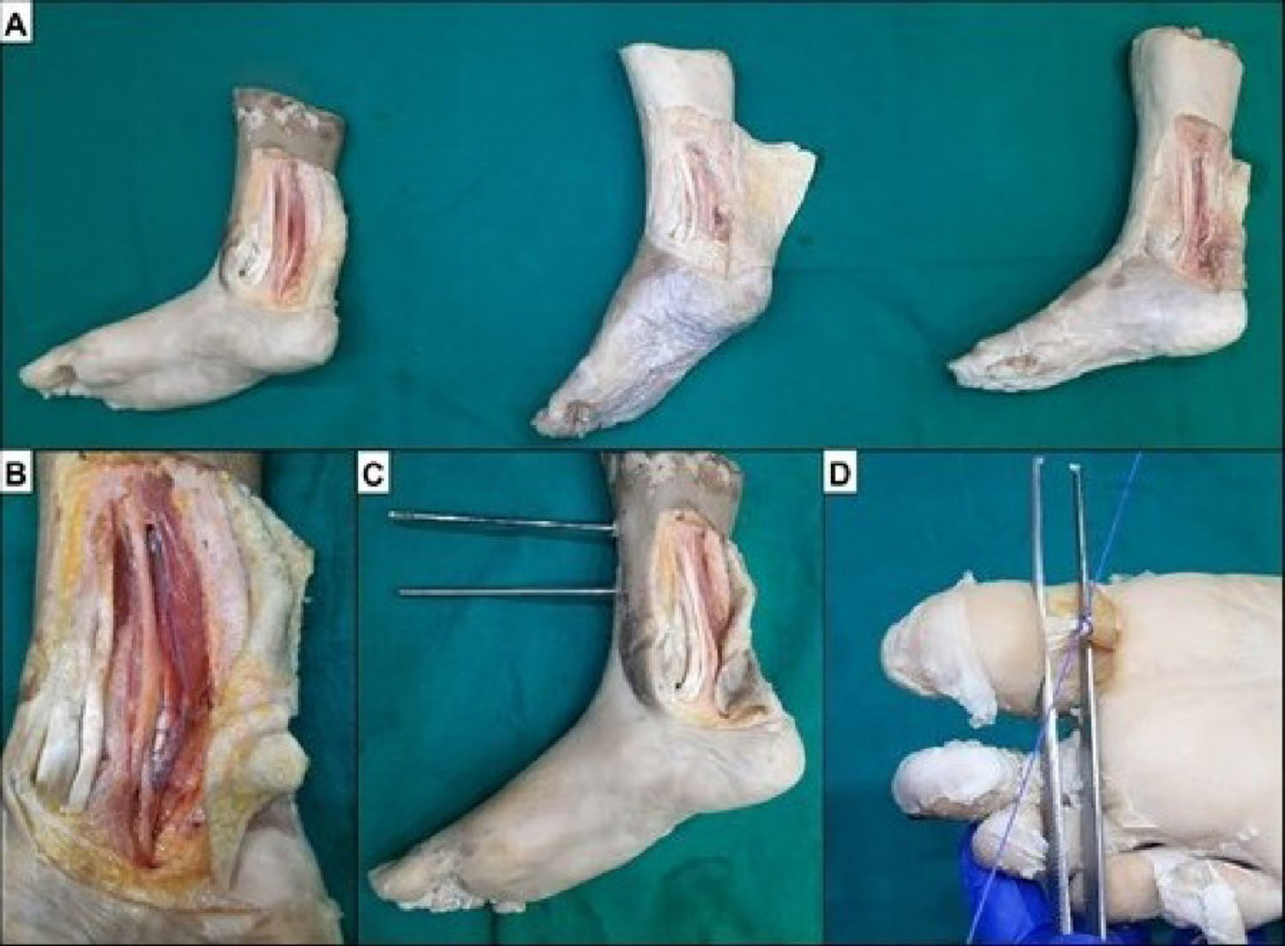
**A** Exposing and isolating the FHL tendon. **B** Passing Krackow sutures through the FHL tendon. **C** Applying a Schanz screw to a cadaver. **D** Isolating the tendon by dissecting distally

**Fig. 2:**
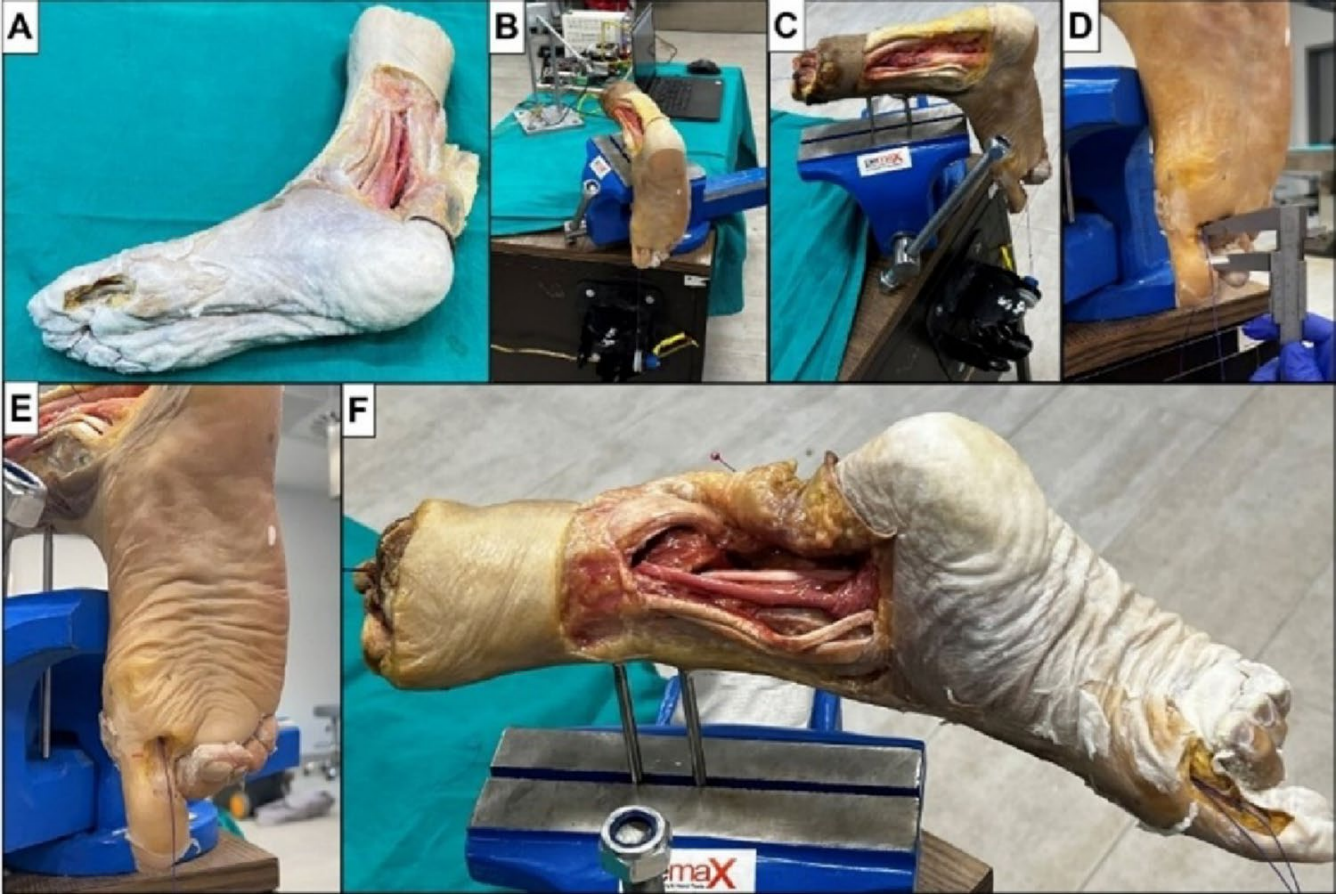
**A** Cadaveric specimen with isolated FHL muscle. **B** Placing the cadaver prepared for measurement into the assembly. **C** Fixing the cadaver using a bench vice. **D** Measurement of the amount of tendon retraction using a caliper. **E** Measurement of the length of retraction during tendon pulling using an encoder. **F** Flexion of the FDL tendon after retraction of the FHL tendon

Foot cadavers were dissected to expose the FHL tendon (Figs. 1A and 2A). Krackow sutures were placed near the musculotendinous junction of the muscle. The sutures were applied in two layers using no. 2 Ethibond thread (Fig. 1B). The Krackow suture technique is designed to provide a secure locking mechanism that distributes tensile loads evenly across the tendon fibers, thereby increasing the pull-out strength and preventing construct failure.

**Fig. 3:**
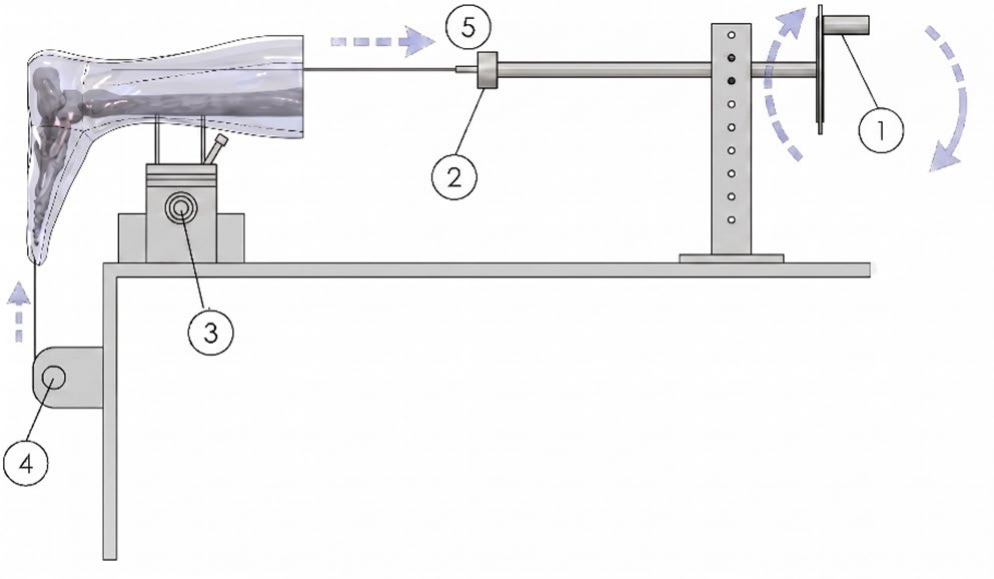
Schematic view of the measurement assembly (1: stretching–pulling assembly; 2: loadcell; 3: sample fixation assembly; and 4: encoder), 5: Direction of pull of FHL

In each of the below-knee cadaveric specimens, Schanz screws were applied to the tibial shaft from the anterior aspect, parallel to the first metatarsal under fluoroscopic guidance. The first screw was placed 10 cm above the sole of the foot, and the second screw was positioned 15 cm above the sole (Fig. 1C). The legs, with the screws in place, were then secured to the prepared device using a bench vice (Figs. 2B F and 3-3). The following procedures were performed on the sole of the foot in sequential order before securing it in the bench vice. The skin was dissected at the distal phalanx of the hallux. The FHL tendon was exposed until the level of the proximal phalanx and first metatarsal head. The distal insertion of the FHL tendon at the distal phalanx was released to simulate a hallux-level laceration. (Fig. 1D).

The severed tendon was sutured using no. 2 Ethibond threads and this suture attached to encoder (Fig. 1D). The purpose of this procedure was to measure the length of the thread or the distance of tendon retraction.

### Measurement assembly

The test assembly is shown in Fig. 3. This system included sample fixation (3), tensile loading (1), loadcell (2), and encoder (4).

Sample-fixing assembly (3): Schanz screws were applied to the tibiae of the amputated feet to fix the feet for stretching and pulling using a bench vice fixed on the table. Before initiating tensile loading, all specimens were standardized with the ankle, hindfoot, hallux, and lesser toes in a neutral position. (Fig. 2B, C, and Fig. 3)

Stretching and pulling assembly (1): A handwheel with a screw mechanism was manually applied at the same speed to create a pulling force (Fig. 3-1). This wheel could move vertically in a slide to adjust the height of the foot and ensure parallelism. Once the foot was fixed to the assembly, tensile force was applied parallel to the long axis of the tibia and aligned with the FHL tendon. The hand wheel moved forward 1.25 mm with each turn, pulling the FHL tendon toward the proximal tibia. The handwheel was rotated at a constant rate, with one complete turn performed every 10 s from the marked starting position. Rotation was continued without interruption until the predefined maximum tensile load of 120 N or construct failure was reached.

Loadcell (2)An S-type loadcell was used to measure the tensile forces applied to the FHL tendon with high precision. This comprised a standard force sensor with force measurement up to 1 kN (kilonewton) (Fig. 3-2).

**Fig. 4:**
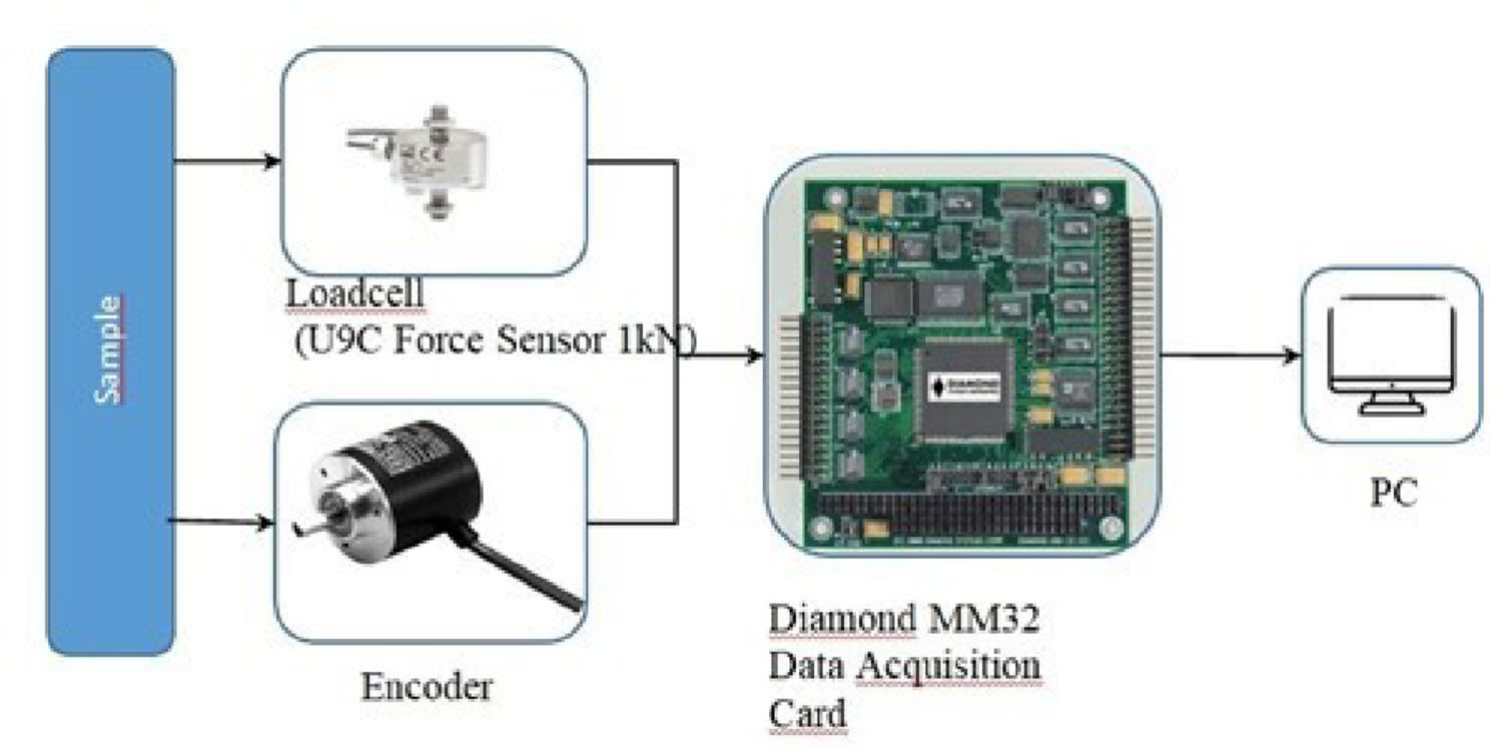
Schematic illustration of the Daq system

Encoder (4) A digital encoder recorded the longitudinal displacement of the tendon in millimeters. The encoder continuously transmitted tendon displacement data, measured in millimeters, to the computer. (Figs. 3 and 4).

**Fig. 5:**
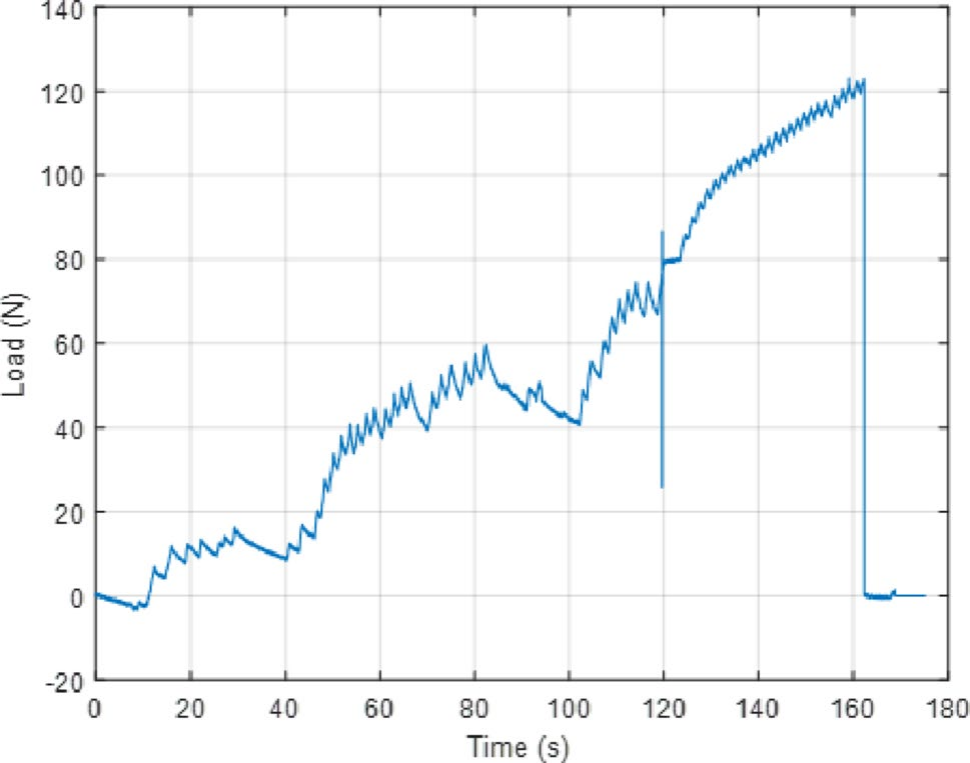
FHL tendon response during tensile loading

Direction of pull: The traction was applied parallel to the anatomical axis of the FHL tendon to simulate the natural direction of tendon retraction during muscle contraction (Figs. 3, 4 and 5).

The last element of the system was the data acquisition (Daq) system. Figure 4 shows a schematic illustration of the data acquisition (DAQ) system.

The tensile force and tissue displacement were measured using the Daq system shown in Fig. 2D. The data were collected with an accuracy of 0.01 in the force measurement. The movement of the tissue was detected using an encoder. The measurements were transferred to the computer simultaneously through the Diamond MM32 card.

### Measurement

Each foot cadaver was secured to the fixation assembly shown in Fig. 2C using Schanz screws. A suture thread was attached to an encoder by tying it to the tendon at the distal phalanx level. Then, the suture thread applied to the musculotendinous junction of the FHL in two layers was connected to the loadcell (2) shown in Fig. 2C. A hand wheel was used to eliminate slack in the thread, bringing it to the starting position. When the system was in the starting position and ready for use, a pulling force was applied to the muscle tendon using the handwheel. The data transferred to the computer were in the form of a force-time graph, with force increasing proportionally with time. Loading was continued until a sudden drop in the force–time curve was observed. This drop was accepted as construct failure and was attributed to FHL muscle rupture, suture failure, or deformation of the surrounding soft tissues. This decrease indicated the mechanical rupture of the muscle, suture rupture, or destruction of the surrounding tissues.

### Preliminary study

Before the final experiments, a preliminary feasibility study was performed to evaluate whether the experimental setup and measurement system could be used for this biomechanical model. For this preliminary assessment, two formalin-fixed lower-limb cadaveric specimens were used. Data obtained from preliminary studies (encoder data) showed that the FHL tendon did not move significantly under a force of 20 N (less than 2.5 mm). For this reason, the FHL muscle of six fresh-frozen cadavers was pulled with forces of approximately 20–60 N using the prepared assembly. The ankle was plantar flexed in all trials with force above 60 N, and the degree of flexion increased as the force approached 120 N. During the final test, a maximum force of approximately 120 N was applied.

Also, according to the manufacturer’s data, a No. 2 Ethibond suture possesses such a high tensile strength (approximately 90–120 N) that it exceeds the maximum force that the Flexor Hallucis Longus (FHL) muscle can generate in vivo. In a 2024 study conducted by Avina Babel et al., the contraction force of the FHL muscle was measured to be approximately 1.18–1.20 kgf during maximum voluntary isometric contraction (MVIC). These values, when converted to Newtons, correspond to approximately 11.77 N (1.20 kg × 9.81 m/s²). The previously reported in vivo FHL force was obtained from non-traumatized individuals during hallux flexion strength testing and does not directly represent the tensile retraction force of a lacerated FHL tendon [12]. Based on the preliminary feasibility observations and the anticipated maximum endurance of the Ethibond suture–tendon construct, the maximum load was set at 120 N to characterize the upper boundary of FHL tendon retraction under controlled supraphysiological conditions.

### Statistical analyses

Statistical analyses were performed using IBM SPSS Statistics (Version 26.0). The categorical data were expressed as frequencies and percentages, and the continuous data were expressed as mean ± standard deviation or median [Q1–Q3] with minimum–maximum values. Owing to the exploratory repeated-measures biomechanical cadaveric design and the limited specimen number, the data were primarily analyzed using descriptive statistics. Since the study did not include a side-to-side comparison design, no such comparative analysis was performed. A priori power analysis using GPower (version 3.1.9.7) indicated that a sample size of ≥ 4 specimens was sufficient to achieve a statistical power (1-β) of 0.82 with an alpha level of 0.05, ensuring the reliability of the findings.

## Results

The main criterion in the study design was to limit the applied tensile load to 120 N, which resulted in one of the following outcomes in each trial: rupture of the FHL, deformation of the muscle, or rupture of the suture (Fig. 5).

Additionally, in all force tests exceeding 60 N (where the FHL was retracted by more than 20 mm), the second, third, fourth, and fifth toes flexed due to the effect of the flexor digitorum longus (FDL) (Fig. 2F).

The median retraction lengths under increasing tensile loads were as follows: 0.22 mm (min–max: 0.18–0.32) at 1 N, 3.40 mm (min–max: 3.20–3.70) at 10 N, 6.90 mm (min–max: 6.40–7.60) at 20 N, 9.65 mm (min–max: 9.50–10.20) at 30 N, 10.85 mm (min–max: 10.60–11.10) at 40 N, 13.40 mm (min–max: 13.10–13.70) at 50 N, and 20.70 mm (min–max: 19.60–21.10) at 60 N. All six cadaveric specimens contributed data from 1 N to 60 N, whereas failure occurred in all specimens between 60 N and 120 N due to Ethibond suture rupture, FHL muscle rupture, or surrounding tissue deformation. The length of muscle retraction distances of the FHL by tensile forces was expressed in millimeters, as shown in Table 1.

**Table 1:** FHL tendon retraction distances under increasing tensile loads

	Group	Median [Q1–Q3] (mm)	Min–max (mm)
Distance	1 N	0.22 [0.19–0.28]	0.18–0.32
	10 N	3.40 [3.35–3.55]	3.20–3.70
	20 N	6.90 [6.40–7.35]	6.40–7.60
	30 N	9.65 [9.58–9.90]	9.50–10.20
	40 N	10.85 [10.75–11.10]	10.60–11.10
	50 N	13.40 [13.25–13.63]	13.10–13.70
	60 N	20.70 [19.83–20.88]	19.60–21.10
	60–120 N	Ethibond or muscle ruptured	

**Fig. 6:**
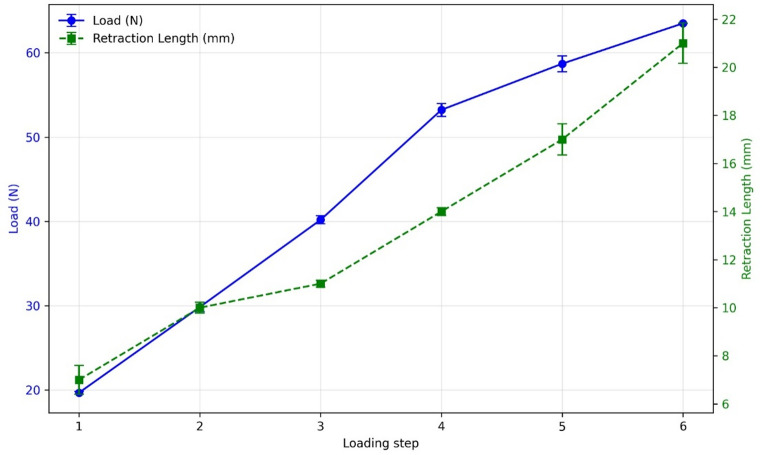
Changes in load and FHL tendon retraction length across loading steps

A progressive increase in applied tensile load.and corresponding FHL tendon retraction was observed across sequential trials. In the first loading step, the mean maximum tensile load was measured as 19.66 ± 0.18 N, with an associated mean retraction length of 7 ± 0.6 mm. The second loading step yielded a tensile strength of 29.83 ± 0.56 N and a retraction length of 10 ± 0.22 mm. This trend continued in the third (40.18 ± 0.48 N; 11 ± 0.14 mm), fourth (53.23 ± 0.77 N; 14 ± 0.16 mm), fifth (58.71 ± 0.95 N; 17 ± 0.65 mm), and sixth (63.50 ± 0.04 N; 21 ± 0.84 mm) loading steps. These changes in load and FHL tendon retraction length across loading steps are shown in Fig. 6.

## Discussion

In this novel study; we found that FHL tendon injuries occurring at the hallux level show slight tendon retraction that allows for primary repair for delayed surgical period.

The study found that, with the combined effect of the FHL and flexor digitorum longus (FDL) muscles, the maximum retraction was approximately 20 mm, resulting in flexion of the other toes and increased plantar flexion of the ankle proportional to the applied force. Compared to Rowley et al. (2023), who measured toe grip force indirectly via a dynamometer on the tendon with a mean of 12.6 ± 4.78 kg, the present study applied direct force to the isolated muscle, explaining the methodological differences and variation in force values between studies [13]. Consequently, the forces applied in Rowley’s study and those in the present study differed significantly due to the variations in technique [13]. In this study, we found that FHL tendon injuries at the hallux level exhibit mild retraction, potentially allowing for primary repair even in delayed cases. Our results revealed that the combined action of the FHL and flexor digitorum longus (FDL) muscles limits the maximum retraction to approximately 20 mm.

To our knowledge, no previous study has directly quantified proximal tendon retraction after complete FHL tendon laceration. In the hand region, flexor tendons retract due to myotonic contraction of the muscles following tendon lacerations [10]. Tendon retraction is particularly observed in tendon injuries located in zones III, IV, and V of the hand [11]. In a study conducted on chronic EHL tendon laceration, findings were presented showing that the tendon was retracted by approximately 3 cm and that the retracted tendon could be repaired using the Z-plasty method [14].

Prolonged time to surgical repair may result in permanent tendon retraction. This condition often necessitates longer proximal surgical incisions and prolongs the operation time due to the need to locate and suture the retracted tendon ends. However, there is still a lack of valid and detailed information regarding flexor tendon injuries and their behavior in the foot region.

**Fig. 7:**
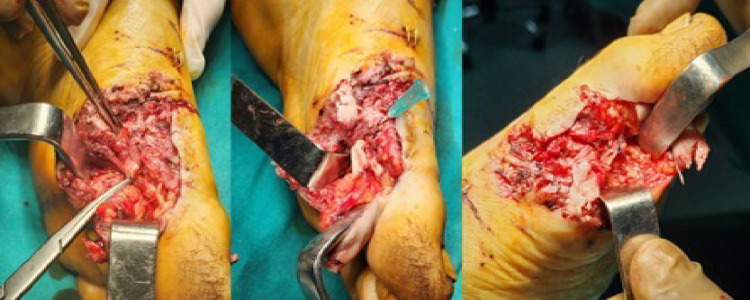
Repair of an FHL rupture

Only three primary FHL laceration repairs have been performed at the acute traumatic metatarsophalangeal joint level in the shortest time since the planning of the study in the orthopedics and traumatology clinic of **********************. In two of these cases, surgical repair was performed within the first 12 h based on the principles of hand flexor tendon surgery and the possibility of FHL retraction. In one patient, surgery was planned and performed on the seventh day of injury due to cerebral hemorrhage (Fig. 7). The surgical exploration showed no retraction of the tendon. The tendon proximal to the incision did not go more proximally than the level of plantar slip and plantar fascia on the sole of the foot. These clinical cases were not included in the formal study cohort and are presented only as illustrative observations. Nevertheless, the delayed repair case raised the clinical question of whether hallux-level FHL tendon lacerations result in clinically relevant proximal tendon retraction, thereby motivating the design of the present cadaveric biomechanical study.

Some studies have shown that the FHL and FDL are connected. Plaas et al. (2013) identified that FHL and FDL were interconnected as variations grouped into four main types and four subtypes. The connections originating from the FHL and directed toward the FDL were observed 2–8 cm distal to the medial malleolus [15]. The FHL fused with the FDL on the sole of the foot and attached to the second, third, and fourth toes via these connections. The connections formed by the fibers originating from the FDL and extending to the FHL were found to be 3.5–7 cm distal to the malleolus medialis. In their study with 20 formalin-fixed cadaveric feet, Beger et al. (2018) reported that Henry’s knot, the intersection site where the FDL tendon crosses over the FHL tendon, is widely used as a surgical landmark during tendon graft harvesting [16]. However, the exact anatomical location of this important region remains controversial. Regarding surgical timing and technique, Lee et al. (2024) emphasize that while primary end-to-end repair remains the gold standard for acute injuries, delayed presentations often necessitate complex reconstruction. However, the minimal retraction observed in our study suggests that the window for primary repair may be wider than previously thought, potentially reducing the need for grafts even in subacute cases. Furthermore, following their technical recommendations, ensuring a 7–10 mm suture purchase from the tendon edge and utilizing at least 2–4 core suture strands can maximize repair strength in these distal FHL lacerations where anatomical tethering is present [17]. Therefore, in the present study, the FHL and FDL moved together at a pulling force of 60–120 N and above, and the other four toes flexed under the effect of the FDL. Thus, given that the FHL tendon demonstrates limited retraction in injuries localized at the hallux level, surgical planning can take other clinical factors and patient-specific conditions into account. In addition, we believe that the combined movement of the FHL and FDL tendons give insight into the literature concerning the ideal positioning of casting during the perioperative period following FHL injuries.

Lee and Elliot (2017) examined the effect of tissue freezing on tendon mechanics and damage mechanisms [18]. They detected no difference between fresh and frozen tendons in any of the macro- and micro-scale parameters, including three freeze-thaw cycles. They reported that the damage mechanisms observed in frozen tendons were also valid for fresh tendons. Therefore, they suggested that the storage method and the number of freeze-thaw cycles should be carefully evaluated and then used for future experiments. The most important factor ensuring data reliability in this study was that each foot-leg cadaver was allowed to reach room temperature only once, with careful consideration given to the force and force/retraction distances of the tendons during this process. Given the limited retraction observed in distal FHL incisions due to these tendinous interconnections, surgical planning can be optimized. The fact that the tendon did not retract more than 20 mm even under significant force suggests that surgeons may encounter less difficulty in locating the proximal stump than previously anticipated in clinical practice.

### Limitations

This study had some limitations, the most important of which was the low number of cadavers. Nowadays, obtaining fresh-frozen cadaveric specimens is challenging. Another limitation was that the system and assembly used were an experimental mechanically activated setup, and the data were recorded electronically. Hence, the system was semi-manual. However, tensile forces were still applied in the contraction direction of the FHL muscle, and the tendon retraction distances could be measured with numerical data. As this is a cadaver study, the fact that muscle tone differs from that of living individuals and that no biological healing response occurs limits the extent to which the findings can be directly applied to clinical practice.

## Conclusions

In conclusion, this study found that the FHL tendon retracts by approximately 2 cm even with the application of maximum force. The retraction distance in FHL lacerations tends to be limited due to the tendinous interconnections between the FHL and FDL. This study may provide guidance for surgical planning and postoperative casting strategies by clarifying FHL biomechanics. However, further clinical research is warranted to correlate these cadaveric findings with in vivo conditions and to fully investigate their clinical implications.

## Data Availability

A paper copy of the database is available at Niğde Ömer Halisdemir University.

## References

[1] Karasick D, Schweitzer ME (1996) The os trigonum syndrome: imaging features. Am J Roentgenol 166(1):125–129. 10.2214/ajr.166.1.857186 8571860 10.2214/ajr.166.1.8571860

[2] Michelson J, Dunn L (2005) Tenosynovitis of the flexor hallucis longus: a clinical study of the spectrum of presentation and treatment. Foot Ankle Int 26(4):291–303. 10.1177/107110070502600405 15829213 10.1177/107110070502600405

[3] Amlang M, Rosenow MC, Friedrich A, Zwipp H, Rammelt S (2012) Direct plantar approach to Henry’s knot for flexor hallucis longus transfer. Foot Ankle Int 33(1):7–13. 10.3113/FAI.2012.0007 22381230 10.3113/FAI.2012.0007

[4] Drake R, Vogl AW, Mitchell AW (2009) Gray’s anatomy for students E-book. Elsevier Health Sciences, Philadelphia

[5] Edama M, Kubo M, Onishi H et al (2016) Anatomical study of toe flexion by flexor hallucis longus. Anat Anz 204:80–85. 10.1016/j.aanat.2015.11.008 10.1016/j.aanat.2015.11.008 26704354

[6] Hyman J, Rodeo SA (2000) Injury and repair of tendons and ligaments. Phys Med Rehabil Clin N Am 11(2):267–288 10810761

[7] Kolettis GJ, Micheli LJ, Klein JD (1996) Release of the flexor hallucis longus tendon in ballet dancers. J Bone Joint Surg Am 78:1386–1390. 10.2106/00004623-199609000-00014 8816655 10.2106/00004623-199609000-00014

[8] Scaduto AA, Cracchiolo A (2000) Lacerations and ruptures of the flexor or extensor hallucis longus tendons. Foot Ankle Clin 5:725–736 11232406

[9] Lin TW, Cardenas L, Soslowsky LJ (2004) Biomechanics of tendon injury and repair. J Biomech 37(6):865–877. 10.1016/j.jbiomech.2003.11.005 15111074 10.1016/j.jbiomech.2003.11.005

[10] Schneider LH, Hunter JM, Norris TR, Nadeau PO (1977) Delayed flexor tendon repair in no man’s land. J Hand Surg Am 2(6):452–455. 10.1016/S0363-5023(77)80026-9 336676 10.1016/s0363-5023(77)80026-9

[11] Sasor SE, Chung KC (2023) Surgical Considerations for Flexor Tendon Repair: Timing and Choice of Repair Technique and Rehabilitation. Hand Clin 39(2):151–163. 10.1016/j.hcl.2022.08.016 37080647 10.1016/j.hcl.2022.08.016

[12] Babel A, Arunmozhi R, Yadav P, Sonone T, Negi R (2024) An observational study to determine the reliability of modified digital dynamometer to check the strength of flexor hallucis longus muscle. SALT J Sci Res Healthc 4(1):67–71

[13] Rowley M, Kurıhara T, Ortiz-Weissberg D, Kulig K (2023) Contributions of flexor hallucis longus and brevis muscles to isometric toe flexor force production. Acta Bioeng Biomech 25(1):91–99. 10.37190/ABB-02222-2023-02 38314582

[14] Gilliéron P, Vautrin M, Stanekova K, Crevoisier X (2025) Z-lengthening plasty of the extensor hallucis longus (EHL) tendon proximal to the retinaculum extensorum to repair a chronic rupture of the distal EHL tendon. Tech Foot Ankle Surg 24(4):10–1097

[15] Plaass C, Abuharbid G, Waizy H et al (2013) Anatomical variations of the flexor hallucis longus and flexor digitorum longus in the chiasma plantare. Foot Ankle Int 34(11):1580–1587. 10.1177/1071100713494780 23788233 10.1177/1071100713494780

[16] Beger O, Elvan Ö, Keskinbora M, Ün B, Uzmansel D, Kurtoğlu Z (2018) Anatomy of master knot of Henry: a morphometric study on cadavers. Acta Orthop Traumatol Turc 52(2):134–142. 10.1016/j.aott.2018.01.001 29366540 10.1016/j.aott.2018.01.001 PMC6136317

[17] Lee SW, Guild TT, Burgesson B, Kwon JY (2024) Tendon lacerations of the foot and ankle: a contemporary review. Foot Ankle Int 46(1):115–125. 10.1177/10711007241292068 39503433 10.1177/10711007241292068

[18] Lee AH, Elliott DM (2017) Freezing does not alter multiscale tendon mechanics and damage mechanisms in tension. Ann N Y Acad Sci 1409(1):85–94. 10.1111/nyas.13460 29068534 10.1111/nyas.13460 PMC6611696

